# Understanding nontuberculous mycobacterial lung disease: it’s been a long time coming

**DOI:** 10.12688/f1000research.9272.1

**Published:** 2016-11-30

**Authors:** David E. Griffith, Timothy R. Aksamit

**Affiliations:** 1 University of Texas Health Science Center at Tyler, Tyler, Texas, USA; 2 Mayo Clinic, Pulmonary Disease and Critical Care Medicine, Rochester, Minnesota, USA

**Keywords:** Nontuberculous, mycobacterial, lung disease

## Abstract

With a surprising predictability, most studies and reviews addressing therapy for nontuberculous mycobacterial (NTM) lung disease either start or end by mentioning the paucity of data from randomized and controlled trials. That is a legitimate criticism for NTM lung disease therapy, but it also somehow seems to influence attitudes toward all aspects of NTM investigation. Certainly the study of NTM diseases in general and NTM lung disease in particular is a recent development. Previously, NTM were viewed as minor, if inconvenient, pathogens similar to *Mycobacterium tuberculosis*. However, over the last three decades, NTM have emerged as increasingly important pathogens that are clearly different compared with tuberculosis. Although there has been frustratingly slow progress in the treatment of NTM diseases, in contrast there has unquestionably been impressive progress in almost every other realm of investigation into NTM disease. Our understanding of NTM lung disease a) pathophysiology, including mechanisms of organism acquisition, b) epidemiology, including estimates of disease prevalence, c) mycobacteriology, including application of molecular laboratory techniques and matrix-assisted laser desorption ionization–time of flight (MALDI–TOF) mass spectrometry, and d) even treatment strategies, including the recognition of innate drug resistance mechanisms, has immeasurably and permanently changed and advanced the landscape for NTM lung disease. It is no longer necessary to apologize for the state of NTM lung disease knowledge and understanding, but rather it is time to recognize the great distance we have travelled over the last 30 years.

## Introduction

In a candid indictment of the state of nontuberculous mycobacterial (NTM) lung disease knowledge, a friend, colleague, and respected NTM disease expert recently commented that he thought we had “learned nothing in the last 30 years” about NTM lung disease. The comment was made in the context of ongoing efforts to revise NTM lung disease treatment guidelines. We suspect his intent was due to frustration with the painfully slow progress of NTM lung disease therapy, the paucity of effective antibiotic agents for NTM, and a lack of randomized treatment trials for determining which of the available under-achieving agents are most effective. We don’t believe he literally meant that “nothing” had been learned about NTM disease in three decades, but we were surprised by the number of our colleagues who also heard his statement and apparently shared his frustration, as demonstrated by their approving nods. Our colleague’s statement stimulated our own sober reflections about where we are and where we have been with NTM lung disease over the last three decades.

In many ways, the evolution of NTM lung disease knowledge and understanding has been a struggle to separate and differentiate NTM pathogens from *Mycobacterium tuberculosis* (MTB). The more we know about NTM respiratory pathogens, the less MTB is a pertinent model for them in the realm of therapy. Unfortunately, disseminating that message to the clinicians caring for NTM disease patients is a slow and frustrating process ^1^.

## Species identification

Thirty years ago, NTM organisms were identified and classified using slow and insensitive phenotypic criteria that relied on colony morphology and patterns of biochemical metabolism ^2, 3^. This classification system was eponymously labeled the Runyon classification system after Dr Ernest H. Runyon, who organized and promoted this important early NTM classification system. Thirty years ago, the total number of recognized NTM species was approximately 40–50 ^4, 5^. Clinically isolated NTM identification rapidly improved in terms of both speed and accuracy with the application of high-performance liquid chromatography (HPLC) and the introduction of molecular laboratory methods, including DNA probes (which were also used for early identification of MTB) and gene sequencing techniques ^6– 9^. Both HPLC and DNA probes had limited utility and were restricted to identification of the most frequently isolated NTM species. NTM species identification rapidly expanded in an unprecedented manner with the widespread application of 16S rRNA gene sequencing, a gene thought to be highly preserved within NTM species ^10^. Largely on the basis of this technique, the number of recognized NTM species has grown to nearly 200. It is also now apparent that the 16S rRNA gene is not as well preserved within species as assumed so that it is unfortunately not always a satisfactory or accurate arbiter of NTM species status ^10, 11^. The process of organism identification through gene sequencing has expanded well beyond 16S rRNA sequencing and become so sophisticated that discriminating between many NTM species requires either multi-gene sequencing or whole-genome sequencing ^10^. Interestingly, it is still not established how much difference in whole-genome sequencing is necessary for species differentiation ^10^. Additionally, molecular laboratory methods have provided not only rapid and accurate identification of clinical NTM isolates but also windows into innate NTM antibiotic resistance mechanisms (see below).

Organism genotyping has also proven to be a useful tool for evaluating environmental niches of NTM, thereby providing insights into routes of NTM pathogen acquisition ^12, 13^. This technique also allows discrimination between true disease relapses and (presumed) reinfection ^14^.

The use of molecular laboratory methods has so completely changed the way we see NTM lung pathogens and disease that the advances have outstripped the ability and capacity of most mycobacterial laboratories to adopt these invaluable techniques. Currently, most mycobacterial laboratories in the U.S. do not utilize the new and sophisticated molecular methods or matrix-assisted laser desorption ionization–time of flight (MALDI–TOF) mass spectrometry so that NTM species identification outside of the most common species requires referral of NTM isolates to a few U.S. reference mycobacteriology laboratories.

## Prevalence

Thirty years ago, the limited understanding of NTM disease epidemiology in the U.S. was based on isolation prevalence of NTM isolates sent to the Centers for Disease Control and Prevention (CDC) for processing ^15, 16^. Pulmonary NTM disease was and still remains a non-reportable disease, so the isolates received by the CDC were submitted voluntarily and serendipitously rather than as part of an organized or comprehensive national survey of NTM isolates. With this poorly defined and random approach, U.S. NTM disease prevalence was estimated to be 1–2 cases/100,000 population ^15^. It is noteworthy that at the time there were few comparable estimates for NTM lung disease in most of the rest of the world.

The major impediment to more accurate NTM lung disease prevalence is the well-recognized observation that, contrary to MTB, a single NTM isolate is not necessarily indicative of active NTM lung disease ^5, 17– 20^. Unlike MTB, simple isolation prevalence of an NTM species from respiratory specimens does not indicate the actual lung disease prevalence associated with that NTM species. Patients must meet diagnostic criteria, which are sometimes difficult to ascertain in retrospect without detailed evaluation of the patient’s medical record. Investigators have begun to surmount this formidable obstacle in two ways. First, a few investigators have performed the tedious and labor-intensive retrospective analysis of patient medical records, which is necessary for accurate NTM case definition ^21– 23^. Second, investigators have utilized other epidemiologic tools and approaches such as querying extensive patient databases, including those from Medicare and large HMOs ^24– 26^. While acquiring data through mandatory NTM disease reporting is still the goal for NTM lung disease epidemiology research, current estimates of NTM lung disease prevalence in the U.S. are now much more reliably informative and suggest that NTM lung disease prevalence may be as high as 50 cases/100,000 population in some demographic groups ^23^. Equally as important, investigators in many parts of the world are using similar innovative approaches so that a clearer picture of global NTM lung disease prevalence is emerging ^26, 27^. Many parts of the developing world remain largely unexplored from an NTM epidemiologic standpoint, but even those areas are becoming more accessible through the expanding use of rapid and accurate tools for TB diagnosis, such as the Xpert MTB/RIF technology ^28– 30^. This technology provides a first approximation of NTM disease prevalence by identifying patients who are acid fast bacilli (AFB) smear positive but nucleic acid amplification negative for TB. Similarly, Xpert MTB/RIF can help differentiate patients with NTM infection after TB therapy from patients with recurrent TB. As this technology spreads, the extent of NTM disease in the developing world will be illuminated further and very likely will prove to be significantly more common than is currently appreciated.

## Pathophysiology

Thirty years ago, almost nothing was known about NTM disease pathophysiology, as it was assumed to be analogous to TB with the exception that NTM lung disease pathogens were known not to be transmitted between humans. It was known that NTM were environmental organisms with environmental niches, including natural water sources ^31^. Some investigators speculated that naturally occurring aerosolization of the organism with subsequent inhalation was the major route of NTM lung pathogen acquisition by humans ^32^.

More recently, there has been repeated demonstration of NTM respiratory pathogens from multiple environmental sources including household or municipal water ^12, 13, 33^. Further, as alluded to previously, through the utilization of organism genotyping techniques, it has also been shown that some patients with NTM lung disease have NTM respiratory isolates that are genotypically identical to NTM isolates from household or municipal water ^13^. These data provide persuasive evidence that household water is the source of NTM respiratory pathogens for some patients with NTM lung disease, especially *Mycobacterium avium* complex (MAC) lung disease associated with bronchiectasis ^13^. The only environmentally identified niche for *Mycobacterium kansasii* is municipal water ^17^ so that household water is the likely source of human infection for this organism as well. In addition to aiding our understanding of NTM lung disease pathophysiology, the recognition of NTM in municipal/household water and the demonstration of NTM acquisition from these sources create opportunities for the development of prevention strategies.

Molecular epidemiology techniques have also recently provided the first evidence consistent with human-to-human transmission of an NTM respiratory pathogen ^11, 34, 35^. The dogma for NTM respiratory pathogens has been that they cannot be acquired from exposure to a patient with NTM lung disease. Recent evidence has emerged consistent with transmission of a *Mycobacterium massiliense* isolate among cystic fibrosis patients. One particularly intriguing aspect of this ongoing research is the identification of *M. massiliense* isolates with a high level of genetic relatedness in cystic fibrosis patients from disparate parts of the world without any known contact among the affected patients. There are still questions that must be addressed before it can be said conclusively that NTM transmission occurs between humans, even in a vulnerable population such as cystic fibrosis sufferers, but the work so far is unquestionably provocative.

Thirty years ago, NTM lung disease was regarded as clinically similar to TB including characteristic TB radiographic manifestations with upper lobe fibrocavitary abnormalities ^4, 5^. It is now well established that while NTM lung disease can present in a manner similar to reactivation TB with upper lobe fibrocavitary changes, in the U.S. it is probably more commonly associated with non-cavitary radiographic changes, especially those associated with bronchiectasis ^17, 36, 37^. From a pathophysiologic perspective, this recognition has changed the way that many NTM experts view the development of NTM lung disease. Specifically, because NTM exposure is universal but NTM disease is relatively rare, it is increasingly accepted that patients require not only exposure to NTM but also likely some type of predisposition, such as the structural lung abnormalities most often associated with bronchiectasis or obstructive lung disease ^38^. This hypothesis has been dubbed the “two-hit” theory of NTM lung disease acquisition. For most patients, therefore, NTM infection is the consequence of an underlying anatomic lung disturbance or abnormality rather than a primary event. The etiology of bronchiectasis for many patients remains elusive, but recent work suggests that for at least some patients with “idiopathic” bronchiectasis, there is probably a polygenic explanation for the presence of bronchiectasis ^39^. This exciting work appears to be a particularly fertile and rapidly expanding area of NTM research. An important and as-yet-unanswered question is can vulnerable populations, such as bronchiectasis patients, avoid or even limit NTM exposure, thereby limiting their risk of acquiring NTM infection?

## Treatment

Thirty years ago, the treatment of NTM respiratory pathogens was based primarily on the principles of TB therapy with a limited armamentarium of anti-TB drugs whose use was more or less guided by *in vitro* susceptibility tests with minimum inhibitory concentration breakpoints developed for MTB ^4, 5, 40^. In one contemporary study from a prominent NTM treatment center, it was suggested that treatment success correlated with the number of anti-TB drugs used in the treatment regimen (up to five or six), including second-line TB drugs such as ethionamide and cycloserine ^41^. There was wide recognition of the limitations of this approach and few data demonstrating successful outcomes with traditional anti-TB medications.

In the mid 1980’s, with the advent of the AIDS epidemic, a new deadly microbe, MAC, emerged ^42– 45^. A real sense of urgency developed to find an effective therapy for MAC as it became, at one point, the most lethal bacterial pathogen for AIDS patients ^45^. Many antibiotic agents and treatment regimens were tried, with newer macrolides/azalides emerging as the cornerstones of effective disseminated MAC therapy and prophylaxis ^46, 47^. Ultimately, the scourge of disseminated MAC was relegated to a rare occurrence by the introduction of highly active antiretroviral therapy, but undeniable progress for effective MAC therapy was already established.

Over the next three decades, subsequent studies confirmed the utility of macrolides/azalides for treating MAC from any site, including the lung ^48– 55^. Therapy for MAC lung disease has unfortunately been relatively stagnant since the widespread adoption of macrolide-/azalide-containing MAC treatment regimens. While treatment outcomes have been generally favorable, it is still all too apparent that MAC treatment success is still lagging behind the predictably and reliably favorable TB treatment outcomes. Unfortunately, many other NTM pathogens such as *Mycobacterium xenopi*, *Mycobacterium malmoense*, *Mycobacterium abscessus*, and *Mycobacterium simiae* remain even more difficult to treat than MAC ^17, 56^.

Over the last 30 years, it has become apparent that a particularly troublesome and frustrating aspect of NTM lung disease therapy is the repeatedly confirmed observation that *in vitro* susceptibility results for a specific antibiotic may not be predictive of treatment success (or failure) with that antibiotic for multiple NTM pathogens ^57, 58^. For MAC, for instance, the only antibiotic agents where *in vitro* susceptibility predicts *in vivo* response are macrolides/azalides and amikacin ^16, 49, 59^. Those factors that are associated with antibiotic resistance not predicted by standard *in vitro* susceptibility criteria such as MICs are referred to as innate or natural drug resistance factors ^57, 58^.

The new molecular laboratory tools have provided avenues for investigating the paradoxical NTM antibiotic resistance and have made us aware of multiple factors possessed by NTM that are associated with innate or natural drug resistance ^57, 58^. As noted, these innate resistance factors may not be reflected in the MIC of the organism for specific drugs. This phenomenon is perhaps the most vexing characteristic of NTM lung disease for clinicians and the area where experience with TB is least helpful. Probably the best-known example of this phenomenon is the inducible macrolide resistance, or *erm*, gene, present in *M. abscessus* subspecies *abscessus* as well as other *M. abscessus* subspecies and other mycobacterial species, such as *M. tuberculosis* ^60^. The activity of this gene can be detected *in vitro* only by pre-incubation of the organism in the presence of macrolide/azalide. While *erm* gene activity is only one mechanism of innate NTM drug resistance, its recognition has been transformative for how we approach patients with *M. abscessus* respiratory disease. Ultimately, the future of NTM lung disease therapy will be guided by recognition of innate antibiotic resistance mechanisms, which are inevitable and unavoidable, and the discovery of ways to overcome them.

Unfortunately, the discussion of antibiotic drug resistance does not end here. Even with the background of innate drug resistance, many NTM including MAC, *M. abscessus* subspecies *abscessus*, and *M. abscessus* subspecies *massiliense* are also subject to acquired mutational drug resistance, a mechanism for acquired drug resistance well known to clinicians who treat TB. For instance, macrolides/azalides must be protected by effective companion drugs in treatment regimens for MAC as the emergence of macrolide resistance through the selection of organisms with a 23S rRNA mutation conferring macrolide/azalide resistance is associated with poor treatment response and overall outcome ^61^. This type of drug resistance is both predictable and avoidable. All of the insights into drug resistance for NTM pathogens have come to light in the last 30 years.

## Future outlook

Unquestionably, many, many weaknesses and gaps in our understanding of NTM lung disease remain. We need markers of disease activity and NTM organism virulence so that we can predict which patients will have progressive NTM lung disease and require therapy ^62^. That type of marker would allow eliminating the confusing and sometimes insensitive and nonspecific NTM disease criteria. Equally important, we need to be able to identify which patients are likely to relapse after successful therapy. Overall, we need more efficient ways to define NTM lung disease. We need better ways to determine NTM lung disease prevalence and ultimately incidence. Making NTM lung disease reportable would go a long way toward alleviating this problem, but without better ways to accurately diagnose NTM lung disease, even universal reporting of cases based on current diagnostic criteria probably still entails considerable inaccuracies. The most pressing need is for new and more effective antimicrobial agents, a process that will be driven by improved understanding of NTM drug resistance mechanisms. We will need new approaches to NTM disease prevention, a process only possible with early identification of patients at risk for developing NTM lung disease and better understanding of NTM environmental niches and the acquisition of NTM by vulnerable individuals.

This brief discussion is far from comprehensive and focuses on only a few selected areas of NTM lung disease advances. The understanding of NTM lung disease is clearly in its infancy, but with the accelerating pace of discovery and knowledge, the list of those aspects we don’t understand will inevitably continue to grow in unanticipated directions. The more we learn, the more and better questions we are able to ask. It is also remarkable that the progress so far has been accomplished largely without extramural funding from national (U.S.) and international funding agencies. A major priority is educating extramural funding sources about the importance of NTM lung disease as a growing international health burden and convincing them that committing research dollars to this field will yield important and widely applicable results. Lastly, we need to convince potential private and government funding sources to provide support for prospective treatment trials so that we can begin to lay the groundwork for future study designs that are necessary for testing new drugs as they are introduced. In that context, there is movement toward trials of new as well as older but untested antibiotics on the parts of pharmaceutical companies and the FDA, but results are still several years away.

## Conclusion

Have we learned nothing in 30 years? It doesn’t look that way to us, and in fact the study of NTM lung disease has been completely transformed in that time in unprecedented and unanticipated ways. Perhaps the most convincing reasons to be optimistic about continued and accelerating progress with NTM lung disease are the proliferation of investigators around the world and the growing number of very talented young investigators in the field. The number of references using “nontuberculous mycobacteria” in English using an Ovid search was 39 in 1985 and 30 years later, 608 in 2015, an increase of almost 15-fold., a clear reflection of the exponential growth in NTM research. While NTM lung disease understanding has been a long time coming, all the progress that has been made and is inevitably still to come will guarantee that it just as surely will be a long time gone ^63^.
